# Expression of a LINE-1 endonuclease variant in gastric cancer: its association with clinicopathological parameters

**DOI:** 10.1186/1471-2407-13-265

**Published:** 2013-05-29

**Authors:** Gangshi Wang, Jie Gao, Haili Huang, Yu Tian, Liyan Xue, Weihua Wang, Weidi You, Hongwei Lian, Xiaojian Duan, Benyan Wu, Mengwei Wang

**Affiliations:** 1 Department of Geriatric Gastroenterology, China PLA General Hospital, Beijing, China; 2 Department of Pathology, China PLA General Hospital, Beijing, China; 3 Department of Geriatric Medicine, China PLA General Hospital, Beijing, China; 4 Department of Pathology, Cancer Institute (Hospital), Peking Union Medical College, Chinese Academy of Medical Sciences, Beijing, China

**Keywords:** Gastric carcinoma, Long interspersed nuclear element-1 endonuclease, GCRG213 protein, Tissue microarray

## Abstract

**Background:**

Long interspersed nuclear element-1 (LINE-1 or L1), the most abundant and only autonomously active family of non-LTR retrotransposons in the human genome, expressed not only in the germ lines but also in somatic tissues. It contributes to genetic instability, aging, and age-related diseases, such as cancer. Our previous study identified in human gastric adenocarcinoma an upregulated transcript GCRG213, which shared 88% homology with human L1 sequence and contained a putative conserved apurinic/apyrimidinic endonucleas1 domain.

**Methods:**

Immunohistochemistry was carried out by using a monoclonal mouse anti-human GCRG213 protein (GCRG213p) antibody produced in our laboratory, on tissue microarray constructed with specimens from 175 gastric adenocarcinoma patients. The correlation between GCRG213p expression and patient clinicopathological parameters was evaluated. GCRG213p expression in gastric cancer cell lines were studied using Western blotting analysis. L1 promoter methylation status of gastric cancer cells was tested using methylation-specific PCR. BLASTP was used at the NCBI Blast server to identify GCRG213p sequence to any alignments in the Protein Data Bank databases.

**Results:**

Most primary gastric cancer, lymph node metastases and gastric intestinal metaplasia glands showed positive GCRG213p immunoreactivity. High GCRG213p immunostaining score in the primary gastric cancer was positively correlated with tumor differentiation (well differentiated, p = 0.001), Lauren’s classification (intestinal type, p < 0.05) and a late age onset of gastric adenocarcinoma (≥65 yrs; p < 0.05). GCRG213p expression has no association with other clinicopathological parameters, including survival. Western blotting analysis of GCRG213p expression in gastric cancer cells indicated that GCRG213p level was higher in gastric cancer cell lines than in human normal gastric epithelium immortalized cell line GES-1. Partial methylation of L1 in gastric cancer cells was confirmed by methylation-specific PCR. BLASTP program analysis revealed that GCRG213p peptide shared 83.0% alignment with the C-terminal region of L1 endonuclease (L1-EN). GCRG213p sequence possesses the important residues that compose the conserved features of L1-EN.

**Conclusions:**

GCRG213p could be a variant of L1-EN, a functional member of L1-EN family. Overexpression of GCRG213p is common in both primary gastric cancer and lymph node metastasis. These findings provide evidence of somatic L1 expression in gastric cancer, and its potential consequences in the form of tumor.

## Background

Long interspersed nuclear element-1 (LINE-1 or L1) is the most abundant and only autonomously active family of non-LTR retrotransposons in the human genome and comprises about 17% of the human genome [1]. The vast majority of the approximately 500,000 total copies of L1 in the human genome are not capable of transposition on their own due to various mechanisms like truncations and inactivating mutations. However, an estimated 80–100 full-length, retrotransposition competent L1s (RC-L1s) are present in a typical diploid human genome, and a small number, termed “hot L1s” exhibit high retrotransposition efficiencies [2].

Human RC-L1s are about 6.5 kb and encode two proteins (ORF1p and ORF2p) required for retrotransposition [3]. ORF1p is a 40-kDa protein with RNA binding [4] and nucleic acid chaperone activities [5]. ORF2p is a 150-kDa protein with endonuclease (L1-EN) [6] and reverse transcriptase (L1-RT) [7] activities. L1 element may integrate into several hundred thousand genomic locations, at a loosely defined consensus site (5′-TTTT/AA-3′), which is nicked by L1-EN [8]. The host limits the spread of such elements by transcriptional and post-transcriptional silencing mechanisms that reduce activity to tolerable levels [9].

L1 expressed not only in the germ lines but also in somatic tissues. It is suggested that even low levels of somatic DNA damage due to L1 activity have the potential to contribute to genetic instability, aging, and age-related diseases, such as cancer [10,11]. L1-ORF1 and ORF2 were upregulated in a variety of malignancies, such as breast cancer, pancreatic cancer and malignant germ cell tumors, etc. [12-14]. L1 hypomethylation was observed in a variety of malignancies such as head and neck, esophageal and stomach cancers, and in premalignant lesions [15,16]. The degree of L1 hypomethylation was also associated with a more advanced stage and poorer prognosis [17,18].

L1-induced influence to cells is not likely limited only to fully active elements. Even L1 elements with defective ORF1 coding regions might make an RNA that splices to express a functional ORF2 with a number of negative consequences for the cell [19]. Many tissues produce translatable spliced ORF2 (SpORF2) transcript [20]. A spliced L1 transcript which includes integrant reverse transcriptase sequence of ORF2, was found to be essential for human hepatoma cell proliferation [21].

Our previous study [22] identified an upregulated transcript, named gastric cancer related gene 213 (GCRG213), in human gastric adenocarcinoma. BLAST analysis of GCRG213 sequence indicated 88% homology with human LI and revealed a putative conserved domain, apurinic/apyrimidinic endonucleas1 (APE), in GCRG213-ORF. Our latest search of the updated GenBank database shows GCRG213-ORF contains a L1-EN conserved domain. This paper reports our present study that has confirmed the GCRG213 protein (GCRG213p) expression in gastric cancer cell lines by using monoclonal mouse anti-GCRG213p antibody produced in our laboratory (Geriatric institute, China PLA General Hospital, Beijing, China). Additionally, immunohistochemistry (IHC) applied on tissue microarray (TMA) was used to evaluate GCRG213p expression in gastric cancer and normal gastric mucosa. Further analyses were performed to see if there is any correlation between GCRG213p expression and clinicopathological parameters and survival.

## Methods

### Patients

Patients who underwent gastrectomy for gastric cancer in PLA general hospital, Beijing, China from 1991 to 2003 were considered candidates for this study. The sampling criteria were as follows: gender (males or females), age (20 years or older); newly diagnosed (incident) gastric carcinoma without prior treatment; diagnosis histologically confirmed; paraffin embedded tumor, paired surrounding non-tumoral gastric mucosa tissues available, with carcinomas metastatic to lymph node if possible; and positive follow-up results at the time of TMA construction. As a result, 175 cases were recruited in this study, comprising 144 men and 31 women (27 ~ 84 years, mean = 58.58 years). Among them, sixty seven cases have carcinomas accompanied with lymph node metastasis, and six cases of formalin-fixed, paraffin-embedded normal gastric tissues were also obtained from non-tumor patients who were operated on because of benign gastric diseases.

Demographic, lifestyle and clinicopathological data for the sample cases were shown in Table 1. All tumors were classified and staged according to the revised guidelines advocated by the International Union against Cancer. The follow-up was made to assess their latest conditions in 2005 by consulting their case documents or through phone calls to patients (or their family members, or family practitioners). A minimal interval of 18 months was adopted, and the median follow-up time for patients who were still alive by the end of 2005 was 46 months (the range was from the minimum 18 to the maximum 129 months). Survival time was calculated from the date of surgery to the date of death or the date last known alive.

**Table 1:** Clinicopathological parameters of gastric cancer patients studied in tissue microarray

	**N(%)**	**GCRG213p negative**	**GCRG213p positive**	**P value**
Gender				
Male	144(82.28%)	31	113	0.111
Female	31(17.72%)	11	20	
Age				
<65 year	104(59.43%)	31	73	0.029
≥65 year	71(40.57%)	11	60	
Smoking				
Yes	45(27.27%)	7	38	0.278
No	120(72.73%)	30	90	
Drinking				
Yes	28(16.97%)	2	26	0.060
No	137(83.03%)	35	102	
Family history of tumor				
Yes	14(8.43%)	2	12	0.639
No	152(91.57%)	36	116	
Tumor location				
Cardiac	48(27.43%)	8	40	0.360
Body	37(21.14%)	10	27	
Antrum	90(51.43%)	24	66	
Tumor size (cm)				
≤1	11(6.29%)	3	8	0.506
1-3	42(24.00%)	13	29	
>3	122(69.71%)	26	96	
Depth of invasion				
T1	46(26.28%)	12	34	0.635
T2	21(12.00%)	7	14	
T3	33(18.86%)	6	27	
T4	75(42.86%)	17	58	
Lymph node involvement				
Yes	75(42.86%)	16	59	0.592
No	100(57.14%)	26	74	
Grade of tumor differentiation				
Well	10(5.71%)	0	10	0.001
Moderate	40(22.86%)	4	36	
Poor	103(58.86%)	27	76	
Signet ring cell	22(12.57%)	11	11	
Tumour stage (TNM)				
0	21(12.00%)	3	18	0.217
Ia	14(8.00%)	6	8	
Ib	41(23.43%)	10	31	
II	60(34.29%)	17	43	
IIIa	39(22.28%)	6	33	
Lauren’s classification				
Intestinal type	153(87.43%)	31	122	0.015
Diffuse type	22(12.57%)	11	11	
Resection margin				
Presence	6(3.43%)	0	6	0.361
Absence	169(96.57%)	42	127	
Microvascular invasion				
Presence	20(11.43%)	5	15	0.911
Absence	155(88.57%)	37	118	
Status				
alive	121(71.18%)	30	91	0.483
dead	49(28.82%)	9	40	
5 year survival				
<5 yr	47(43.93%)	12	48	0.871
> = 5 yr	60(50.07%)	10	37	

Permission was given by the ethical committee of the PLA General Hospital, Beijing, China to use the tissue and the data for this project. Informed consent was also obtained from the patients.

### Tissue microarray construction, GCRG213p immunohistochemical staining and assessment

The tissue microarrays (TMAs) were constructed as described previously [23]. From the samples available, six tissue array blocks were prepared, each containing 30 cases with tumor, normal and lymph node tissues if available.

Antigen retrieval of TMA slides, and formalin-fixed, paraffin-embedded tissue sections were performed by pressure cooker treatment for 10 min in an antigen retrieval solution (10 mmol/L sodium citrate buffer, pH 6.0). Monoclonal mouse anti human GCRG213p antibody, which was produced in our laboratory [24], was added at a dilution of 1:200 and incubated for 2 hr at room temperature. The slides were then incubated for 1 h in secondary antibody. An EnVision kit (Dako, Carpinteria, CA, USA) was used to visualize antibody binding, and slides were subsequently counterstained with hematoxylin. A PBS-only staining sample was used as a background control.

Specific yellow-brown immunostaining for GCRG213p was exclusively located in the cytoplasm. It was scored independently and in a blinded manner by two investigators (GJ and XL). The inter-observer disagreements (approximately 6% of total cases) were reviewed for a second time, followed by a conclusive judgment by both observers. Formal scoring was subsequently carried out by one investigator (WG). The staining intensity was categorized as 0 (absent), 1 (weak), 2 (moderate), and 3 (strong). The percentage of positively stained cells was scored as 0 (0% positive), 1 (1-30%), 2 (31-60%), and 3 (>60%). Combined assessing of staining intensity and extension was used to evaluate GCRG213p expression. The minimum score when summed (intensity + extension) was 0, and the maximum was 6 [25]. Overall score of ≥2 was deemed GCRG213p positive.

### Western blotting analysis

Gastric cancer cell lines including SGC-7901, BGC-823 and human normal gastric epithelium immortalized cell line GES-1 were cultured, collected and lysed with the RIPA buffer on ice before being subjected to Western blotting analysis. The protein concentration was detected by the Bradford method with BSA (Sigma-Aldrich) as the standard. Equal amounts of cell extract (40 μg) were subjected to 8% SDS-PAGE and transferred to polyvinylidene difluoride membrane (Bio-Rad) for antibody blotting. The membrane was then blocked, incubated with mouse anti-GCRG213p antibody (1:500) for 2 hr at room temperature, followed by incubation with a horseradish peroxidase–conjugated secondary antibody for 1 hr at room temperature. The signal was visualized with an enhanced chemiluminescence detection reagent. The mouse anti–β-actin antibody (1: 5,000, Sigma) was detected simultaneously as a loading control.

### Detection of methylation status of LINE-1 with MSP

Validation of LINE-1 methylation status was carried out using methylation-specific PCR (MSP). Total DNA of SGC-7901, BGC-823, MGC803 and GES-1 cells was extracted according to the instructions of DNA extraction kit. The DNA was bisulfite modified as described previously [26] for 16 hr at 50°C. Bisulfite-converted DNA was PCR-amplified using Taq polymerase (Invitrogen, USA). Primers used were [27]:

LINEa: unmethylated LINE-1 forward primer, TGTGTGTGAGTTGAAGTAGGGT;

LINEb: unmethylated LINE-1 reverse primer, ACCCAATTTTCCAAATACAACCATCA;

LINEc: methylated LINE-1 forward primer, CGCGAGTCGAAGTAGGGC;

LINEd: methylated LINE-1 reverse primer, ACCCGATTTTCCAAATACGACCG.

PCR amplification conditions were: 4°C,5 min; 94°C,30s,58°C,45s,72°C,61s,40 cycles; 72°C,10 min. The resulting PCR products were visualized on a 2% agarose gel. Methylated DNA Standards from Zymo Research were used as positive control, double distilled water as negative control.

### Sequence similarity and conserved domain search

PSI-Blast was used at the NCBI Blast server [28] (search date: 20^th^ January 2012) to identify GCRG213p sequence to any alignments in the Protein Data Bank databases including all non-redundant GenBank CDS translations, PDB, SwissProt, PIR and PRF. It was performed with a standard initial BLASTP search.

### Statistical analysis

Chi-square test or fisher’s exact probability was employed to evaluate the relationship between GCRG213p expression and clinicopathological variables. Overall survival was examined by GCRG213p expression with Kaplan-Meier curves. All p values were two sided and considered statistically significant if p < 0.05. Statistical analysis was performed using SPSS 13.0 for Windows (SPSS Inc., Chicago, IL).

## Results

### GCRG213p expression patterns in normal gastric mucosa and gastric adenocarcinoma

**Figure 1:**
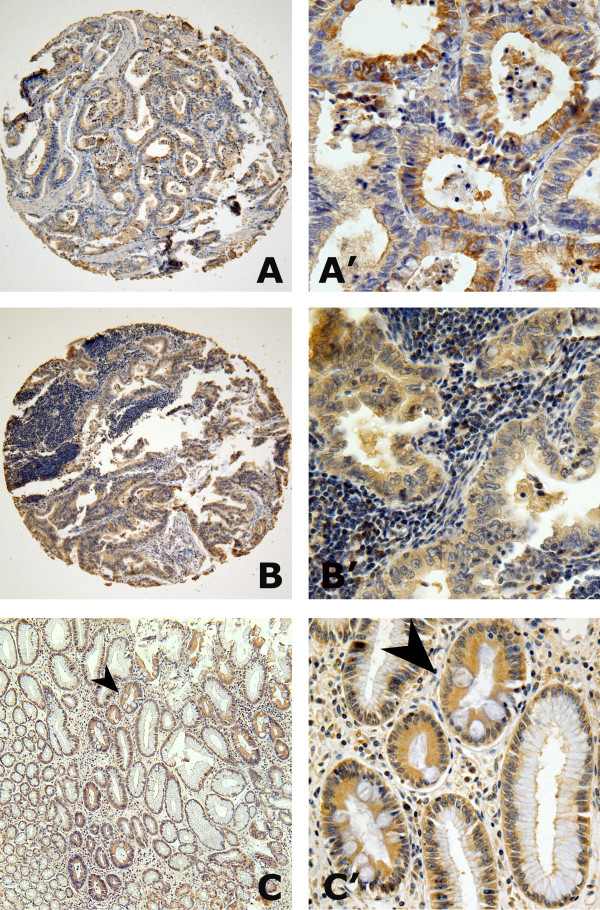
**GCRG213p expression in human malignant and non-malignant gastric mucosa.** Both (**A**) primary gastric adenocarcinoma (100×) and (**B**) adenocarcinoma invaded into the lymph node (100×) showed positive GCRG213p stainging in the cytoplasm; (**A’**) and (**B’**) demonstrated the higher magnification (400×) from the area in (**A**) and (**B**). (**C**) In the non-malignant gastric epithelia, positive staining was observed in the basal membrane but not in the cytoplasm; GCRG213p immunoreactivity was detected in the cytoplasm of gastric intestinal metaplasia glands (arrowed) (100×); (**C’**) demonstrated the higher magnification (400×) from the area in (**C**).

Distinct GCRG213p staining was observed in primary gastric adenocarcinomas, lymph node metastasis tumors, and non-tumoral gastric mucosa (Figure 1). In the non-tumoral gastric mucosa from esopheageal-gastric junction and antrum area, the cytoplasm of differentiated surface epithelia and mucosal glands showed negative staining, but positive GCRG213p staining was observed in the basal membrane of the normal glands. 133 of 175 primary gastric adenocarcinomas, and 51 of 67 tumors metastatic to lymph node showed positive GCRG213p immunoreactivity, which was located in the cytoplasm of the carcinoma cells.

According to the Lauren classification, gastric cancer is devided into two histological types: intestinal-type or diffuse-type. We have 153 intestinal-type cases and 22 diffuse-tye cases in this study. Among the 153 intestinal-type cases, 34 intestinal metaplasia and 28 intraepithelial neoplasia samples adjecent to the cancer were identified. The age (year, mean ± SD) of the intestinal-type group with intestinal metaplasia (59.67 ± 12.30) was older than the diffuse-type group (51.05 ± 11.29) (p = 0.004). 31 of 34 intestinal metaplasia, and 27 of 28 low-/high- grade intraepithelial neoplasia showed GCRG213p immunoreactivity. Most of the positive cases showed mild to moderate staining (Table 2).

**Table 2:** GCRG213 expression in gastric tissue samples

**Groups**	**Number**	**GCRG213 expression**				
		**Overall score**^**‡**^				**PR**^**§**^**(%)**
		**0-1**	**2-3**	**4-5**	**6**	
non-tumoral gastric epithelia	112	112	0	0	0	0.00
Intestinal metaplasia	34	3	18	11	2	91.18 ^*^
Low-/high-grade intraepithelial neoplasia	28	1	17	10	0	96.43 ^*^
adenocarcinoma without LNM ^†^	100	26	50	21	3	74.00 ^*^
adenocarcinoma with LNM	75	16	40	16	3	78.67 ^*^
tumor metastatic to lymph node	67	16	42	8	1	76.12 ^*^

^†^*LNM* Lymph node metastasis; ^‡^ Overall score calculated as (intensity score) plus (percent cells positive score) as described in methods; ^§^*PR* Positive rate.

^*^ Compared with non-tumoral gastric mucosa, p < 0.001.

### Association between GCRG213 expression and clinicopathological parameters

**Figure 2:**
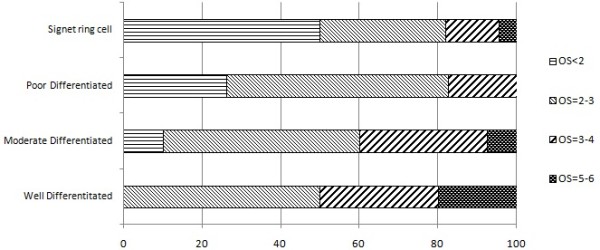
**Correlation of GCRG213p immunostaining score in primary gastric cancer with tumor differentiation.** Overall Score (OS) of immunostaining is higher in the well-differentiated cancer than that in the poor-differentiated (p = 0.001).

High GCRG213p immunostaining score in the primary gastric cancer was positively correlated with tumor differentiation (p = 0.001) (Figure 2). Meanwhile, GCRG213p expression was correlated with Lauren’s classification (p < 0.05). The percentage of positive GCRG213p staining in the aged group (> = 65 yr) was higher than that in the non-aged group (<65 yr) (60/71 = 84.51% vs. 73/104 = 70.19%, p < 0.05). However, GCRG213p expression was not found to be associated with other clinicopathological parameters as those listed in Table 1 (p > 0.05). In the 67 patients with both the primary tumor and lymph node metastatic tumor specimens, GCRG213p was positive in 51 lymph node metastasis specimens and 52 primary tumor specimens (76.12% vs 77.61%), respectively, which indicates that elevated GCRG213p expression in lymph node metastatic tumor is concordant with GCRG213p expression in primary gastric carcinoma. GCRG213p expression in lymph node metastases was also associated with grades of tumor differentiation (p = 0.015), but not with other clinicopathological parameters (p >0.05).

### Relation of GCRG213 expression with survival

Follow-up information was available on 175 gastric cancer patients for periods ranging from 18 months to 14 years. Overall survival rates are as follows: 91.42% (1 year), 78.28%% (2 years), 56.57% (3 years), 39.43% (4 years), and 23.43% (5 years). The median survival rate is 41 months.

Based on GCRG213p expression in primary tumors, there was no significant difference in survival between patients in the GCRG213p negative category compared with the GCRG213 positive category (*X*^*2*^ = 2.072, p = 0.558). Similarly, no correlation was observed between GCRG213p expression in lymph node metastasis and survival (*X*^*2*^ = 3.272, p = 0.195).

### GCRG213p expression in malignant and normal gastric mucosal cell lines

**Figure 3:**
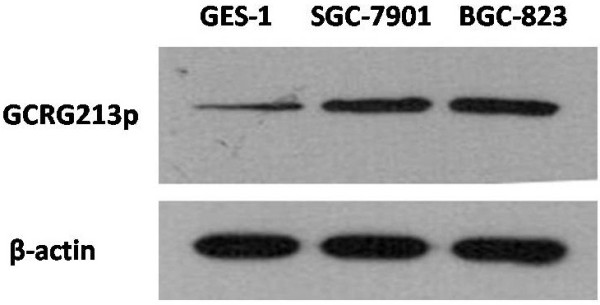
**Western blotting analysis of whole cell extract from gastric malignant and non-malignant cells using anti-GCRG213p antibody.** Cells express GCRG213p with protein bands of 35-kDa. 1:GES-1,2:SGC-7901;3:BGC-823. The filter was reprobed with anti-β-actin antibody to control for equal loading (bottom panel).

Western blotting assays were performed on gastric cancer cells including SGC-7901, BGC-823 and non-malignant gastric mucosal cell line GES-1, in order to further validate the differential expression of GCRG213p. Protein bands of about 35 kDa were identified. Three cell lines tested expressed GCRG213p at different levels. GCRG213p level was found higher in the cancer cell lines than in GES-1 (Figure 3). This finding matches with the IHC result reported in this study, i.e., GCRG213p was found overexpressed in gastric cancer.

### Methylation-specific PCR analysis of LINE-1

**Figure 4:**
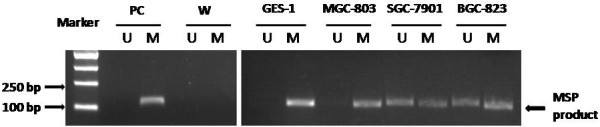
**Methylation-specific PCR (MSP) analysis of *LINE-1*.** Agarose gel electrophoresis of MSP products. M: product amplified with methylated-specific primer; U: product amplified with unmethylated-specific primer. PC: positive control, human methylated DNA standard; W: double distilled water control. Note that the GES-1 samples solely show methylated PCR products, while the MGC-803, SGC-7901 and BGC-823 samples show both methylated and unmethylated PCR products.

Methylation-specific PCR (MSP) analyses were performed on gastric cancer cells and non-malignant gastric mucosal cell line GES-1, in order to test the L1 promoter methylation status in these cell lines. Apart from gastric cancer cell lines SGC-7901 and BGC-823, we also studied gastric cancer cell line MGC-823, for purpose of providing more information about L1 methylation in gastric cancer cell lines. The PCR products amplified with methylated-specific primers (MSPM) and unmethylated-specific primers (MSPU) were 116-bp and 111-bp, respectively. In GES-1 cells, PCR product was amplified with MSPM, but not with MSPU, suggesting that L1 promoters in GES-1 cells underwent complete methylation; In SGC-7901 cells, BGC-823 cells and MGC-803 cells, the corresponding bands can be amplified with both MSPM and MSPU, indicative of partial methylation (Figure 4).

### Bioinformatic identification of GCRG213p as a member of L1-EN family

BLASTP program analysis, as mentioned above, revealed that GCRG213p peptide shared 83.0% alignment with the C-terminal region of L1-EN. Conserved Domain Search of GCRG213p sequence in the Conserved Domain Database of NCBI hits the large exonuclease/endonuclease/phosphatase (EEP) superfamily, including endonuclease domain of the non-LTR retrotransposon LINE-1, exonuclease III (ExoIII)-like apurinic/apyrimidinic (AP) endonucleases, etc.

**Figure 5:**
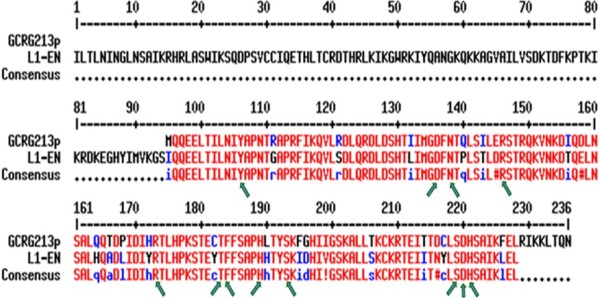
**Alignment of GCRG213p and L1-EN.** Red font represents high consensus, blue or black font represents low consensus. GCRG213p contained residues (arrowed) that are important for the endonuclease activity of L1-EN.

Further analysis using BLASTP indicates that there are in GCRG213p sequence some residues which are important for the conserved features of L1-EN, such as putative phosphate binding site [ion binding site] (Y115/N145/H230), putative metal binding site [ion binding site] (D229) and putative catalytic site [active site] (Y115/D145/N147/D229/H230) (Figure 5).

## Discussion

It is clear that L1 retrotransposition events have occurred in somatic and germ cells. Despite the fact that full length L1 mRNAs are certainly the dominant source of L1 retrotransposition, endogenous L1 ORF2 splice variants with potential biological relevance are found expressed in different somatic tissues. Belancio etc. [20] detected SpORF2 transcript in various adult human tissues and the expression of SpORF2 mRNA exhibits tissue-specific variation. Thus, L1 function is unlikely to be limited only to fully active elements. The SpORF2 can also produce functional ORF2 protein. Most non-LTR retrotransposons are APE-type non-LTR retrotransposons [29]. The crystal structure analyses of the human L1-EN indicate that it is a prototype for AP-like retrotransposon encoded endonucleases, which nick DNA with variable specificity and are responsible for millions of retrotransposon insertions in eukaryotic genomes [30]. Both APE1 and L1-EN belong to the large EEP superfamily that shares the same protein scaffold and the same catalytic residues.

Considering the fact that GCRG213p shares high sequence alignments with L1-EN, and possesses conserved residues which are crucial for L1-EN phosphate binding, metal binding and catalytic activity, we propose that GCRG213 is a spliced L1-ORF2. GCRG213p could be a functional member of L1-EN family, a variant of L1-EN.

The overexpression of L1 in tumors has been widely presented [12-14]. The cellular endogenous L1-RT emerges as a constitutive functional component in tumorigenic cells characterized by a high proliferation and a low differentiation status [31,32]. However, there are few detailed surveys of L1-EN in human tumor. Ergun etc. used anti-L1-EN antibody to detect the L1-ORF2 in adult human tissues, they found strong ORF2p expression in endothelial cells of blood vessels in normal human prostate, urinary bladder and testicular tissue, but no ORF2p expression was observed in the cancer cells studied [33]. Oricchio etc. [34] reported that stable L1 RNA interference could reduce proliferation and promote differentiation in A-375 melanoma cells. They also observed a reduction of L1-ORF2 protein. But the researchers have not identified whether the proliferation effects were from L1-EN and/or L1-RT. To date, there is no report found about the role of L1-EN on cell proliferation and differentiation. In this study, we reconfirmed that GCRG213p expression was elevated in gastric cancer at protein level, both in the cell model and the tissue sections studied, which is consistent with our previous study results at mRNA level [22]. L1 undergo hypomethylation in a variety of tumor tissues, including gastric cancer [35]. Shigaki etc. [36] used bisulfite-pyrosequencing technology to quantify L1 methylation status in resected gastric cancer specimens. They found that patients underwent L1 hypomethylation experienced significantly shorter overall survival than those with hypermethylation. We found in this study that L1 was completely methylated in normal gastric mucosa cell line GES-1, but was partially methylated in gastric cancer cell lines. These results correlate with the GCRG213p expression pattern in gastric cancer cell lines, thus, provide more evidence regarding the nature of the GCRG213 peptide. However, we could not identify a correlation between GCRG213p expression and survival in this study. Intraepithelial neoplasia is believed to be a key step of malignant transformation of gastric adenocarcinoma, overexpression of GCRG213p in these glands implied a potential role of GCRG213p in gastric oncogenesis. The high immunostaining score of GCRG213p in well-differentiated gastric cancer indicated that it might be involved in gastric cancer differentiation.

While chronic atrophic gastritis is believed to be an age-related entity, intestinal metaplasia was considered evidence of atrophic gastritis, since specialized glands had been replaced by intestinal crypts, so intestinal metaplasia was considered as age-related. Age plays an important factor governing the development of gastric intestinal metaplasia, and subjects with gastric intestinal metaplasia were significantly older than those without metaplasia [37]. Intestinal metaplasia glands displayed extensive GCRG213p immunostaining in this study. Meanwhile, we observed that the positive rate of GCRG213p in gastric cancer tissues in the aged group was higher than that in the non-aged group. These might imply that GCRG213p is associated with gastric mucosa aging and age-related entities. There is no study on the association between L1 expression and age at present, but average L1 methylation did not vary over time [38,39]. It is believed that nicking of genomic DNA by the L1-EN can induce cell toxicity, which results in cell cycle arrest, apoptosis or senescence. Expression of exogenous full-length L1 and SpORF2 in normal human fibroblasts, cancer cells and adult stem cells have led to detectable DNA damage and resulted in the senescence-like phenotype [20]. Thus, we suspect that accumulated activity of GCRG213p, a variant of L1-EN, may represent a potential source for age-related cellular transformation, which ultimately contributes to the cell senescence, or oppositely, to an out-of-controlled (malignant) cellular proliferation.

## Conclusions

In conclusion, it is found that overexpression of GCRG213p, a variant of L1-EN, is common in both primary gastric cancer and lymph node metastasis. There is a correlation between GCRG213p expression and tumor differentiation (well-differentiated). No correlation was found with other clinicopathological parameters. GCRG213p expression pattern in the aged group and intestinal metasplasia implied its possible role in gastric mucosa senescence and age-related entities, which deserves further exploration. The data we present usefully contribute to our knowledge of CGRG213p for human normal gastric mucosa and malignancies. These findings provide evidence of somatic L1 expression in gastric cancer, and shed light on its potential consequences in the form of tumor.

## Abbreviations

APE: Apurinic/apyrimidinic endonucleas1; EEP: Exonuclease/endonuclease/phosphatase; GCRG213: Gastric cancer related gene 213; GCRG213p: GCRG213 protein; IHC: Immunohistochemistry; LINE-1 or L1: Long interspersed nuclear element-1; L1-EN: Long interspersed nuclear element-1 endonuclease; L1-RT: Reverse transcriptase; MSP: Methylation-specific PCR; ORF: Open reading frame; RC-L1s: Retrotransposition competent L1s; SpORF2: Spliced ORF2; TMA: Tissue microarray.

## Competing interests

The authors declare that they have no competing interests.

## Authors’ contributions

WG carried out the immunohistochemical studies and the sequence alignment, participated in the collection of clinicopathological data, performed the statistical analysis and drafted the manuscript. HH and WW carried out the tissue array construction and participated in the collection of clinicopathological data. GJ and XL carried out the immunohistochemical staining assessment. TY carried out the Western blotting analysis. YW carried out the immunohistochemical studies. LY and DX carried out the methylation status analysis. WB and WM conceived of the study, and participated in its design and coordination and helped to draft the manuscript. All authors read and approved the final manuscript.

## Pre-publication history

The pre-publication history for this paper can be accessed here:

http://www.biomedcentral.com/1471-2407/13/265/prepub
